# Venous Congestive Ischemic Colitis After Sigmoid Colectomy: A Case Report

**DOI:** 10.7759/cureus.53880

**Published:** 2024-02-08

**Authors:** Naoki Ishimaru, Takashi Tagami, Kohei Takayasu

**Affiliations:** 1 Department of Surgery and Emergency Medicine, Suwa Central Hospital, Nagano, JPN; 2 Department of Emergency and Critical Care Medicine, Nippon Medical School Musashikosugi Hospital, Kawasaki, JPN; 3 Department of Surgery, Suwa Central Hospital, Nagano, JPN

**Keywords:** colorectal surgery, inferior mesenteric vein, mesenteric artery, venous congestion, ischemic colitis

## Abstract

Venous congestion is a possible cause of ischemic colitis following colorectal surgery. As such, congestive ischemic colitis should be considered in such cases where the mesenteric artery is preserved. Herein, we describe the case of a 73-year-old man who presented to the hospital with a two-week history of difficult defecation and frequent mucous stools and was subsequently diagnosed with refractory ischemic enterocolitis due to venous congestion. The patient had undergone resection of the sigmoid colon cancer with preservation of the inferior mesenteric artery 11 months before presentation. Contrast-enhanced abdominal computed tomography revealed edematous wall thickening on the anal side of the anastomosis. A colonoscopy revealed a normal mucosa extending from the anastomosis to the descending colon; however, mucosal swelling, erythema, and erosion were observed on the rectal side of the anastomosis. Based on these findings, he was diagnosed with ischemic colitis. After two months of ineffective conservative treatment, the patient underwent surgery. Ischemic colitis was diagnosed as venous congestion based on the histopathological examination. Preservation of the mesenteric artery may result in ischemic colitis due to an imbalance between the arterial and venous blood flow. Chronic ischemic colitis due to venous congestion should be considered in cases of mesenteric artery preservation to reduce anastomotic leakage.

## Introduction

In colorectal cancer surgery, it is crucial to perform adequate lymph node dissection and prevent complications such as anastomotic leakage and ischemic colitis. Therefore, the optimal mechanisms of ligation and preservation of the mesenteric arteries remain controversial [1].

Most cases of ischemic colitis following colorectal surgery are associated with decreased arterial blood flow [2]. Although ischemic colitis has been known to occur due to venous congestion following colorectal cancer surgery, published data are limited. Nevertheless, it is known that an imbalance between the arterial and venous blood flow in the remaining colon after colorectal surgery may cause ischemic colitis.

Herein, we report an unusual case of chronic ischemic colitis due to venous congestion following surgery for sigmoid colon cancer.

## Case presentation

A 73-year-old man with type 2 diabetes presented to our hospital with a week-long history of difficulty defecating and frequent mucous stools. The patient had no fever or abdominal pain. However, he had undergone resection of the sigmoid colon cancer at the junction with the descending colon 11 months before presentation. The patient had further been treated with D3 lymph node dissection with preservation of the inferior mesenteric artery and functional end-to-end anastomosis. Histopathological examination of the tumor revealed well-differentiated stage IIA. No adjuvant therapy was administered.

**Figure 1:**
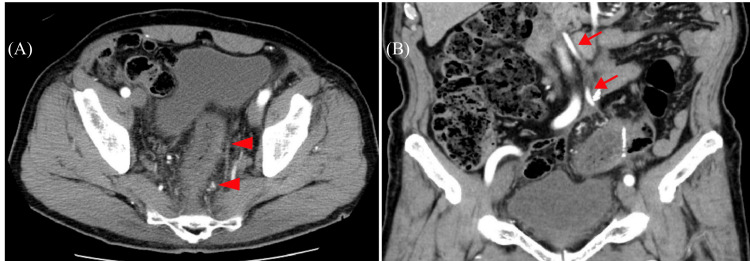
Contrast-enhanced computed tomography. (A) An edematous lesion and thickening (arrowhead) on the anal side of the anastomosis. (B) The inferior mesenteric artery (arrow) is not occluded.

**Figure 2:**
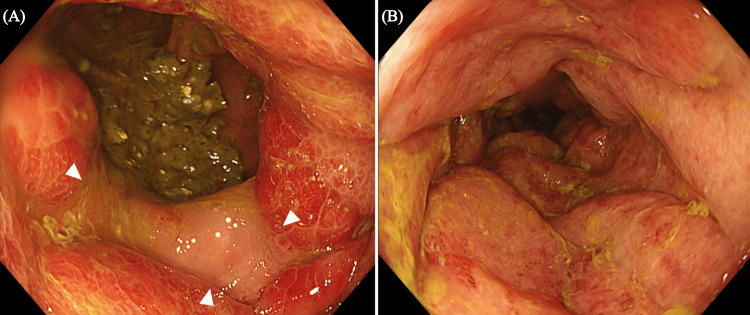
Colonoscopy. (A) Normal mucosa (arrowhead) from the anastomosis to the descending colon. (B) The mucosal swelling, erythema, and erosions were observed on the rectal side from the anastomosis.

Laboratory examinations revealed a white cell count of 12.7 x 10^9^/L (normal range 3.5 x 10^9^ to 9.1 x 10^9^/L) and a C-reactive protein level of 7.2 mg/dL (normal range 0-3 mg/dL). Contrast-enhanced abdominal computed tomography (CT) revealed an edematous wall thickening on the anal side of the anastomosis. In addition, an increased CT value indicating panniculitis was observed surrounding the large intestinal tract (Figure 1). The inferior mesenteric artery was not occluded. A colonoscopy revealed normal mucosa extending from the anastomosis to the descending colon; however, mucosal swelling, erythema, and erosion were observed on the rectal side of the anastomosis (Figure 2). Based on the patient's complaints and clinical examination findings of elevated inflammatory markers, as well as abdominal contrast CT and colonoscopy findings, the patient was finally diagnosed with ischemic colitis.

The patient was hospitalized, and fasting with total parenteral nutrition was initiated. Vasodilators, antibiotics, and steroids were also administered. When the abdominal findings improved, he resumed eating. The patient was readmitted to the hospital two times over the following two months. However, ischemic colitis did not improve, and surgery was ultimately performed.

**Figure 3:**
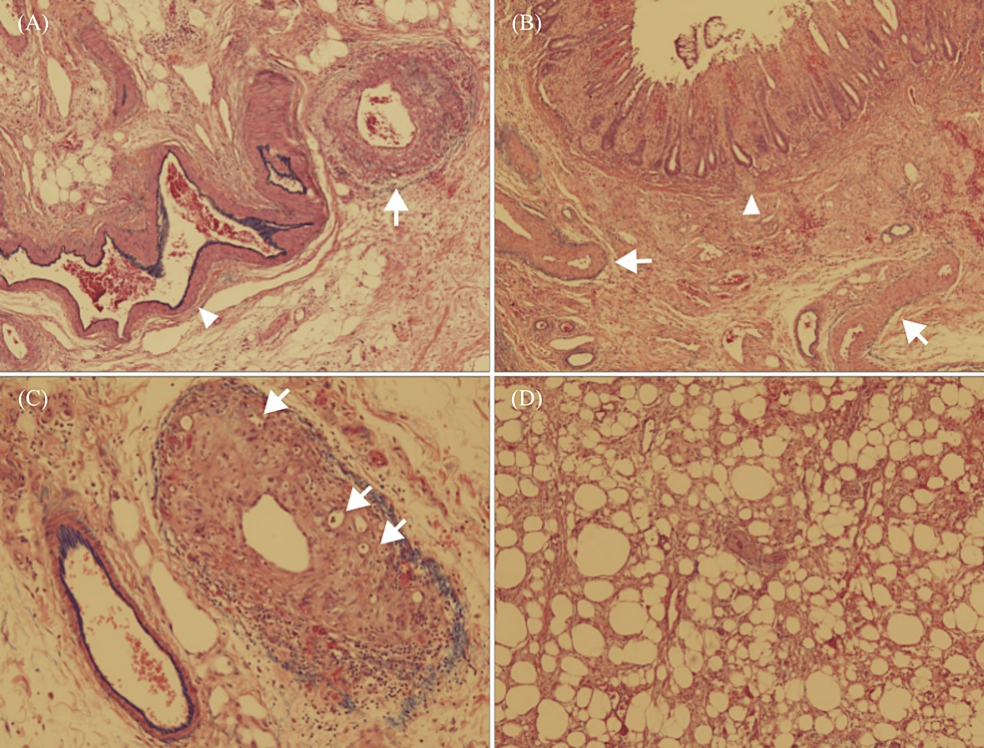
Microscopic pathological findings. (A) A narrowing of the venous lumen with marked thickening of the intima (arrow) can be observed in the subserosa. The arteries (arrowhead) do not show such intimal thickening (Victoria blue hematoxylin and eosin stain, original magnification ×40); (B) intimal vein thickening (arrow) extending from the submucosa to the muscularis propria and the submucosa. The mucosa shows erosion (arrowhead) (Victoria blue hematoxylin and eosin stain, original magnification ×40); (C) sclerotic veins and thrombi (arrow) are present in the submucosa (Victoria blue hematoxylin and eosin stain, original magnification ×100); (D) in the subserosa, macrophage infiltration and fibrosis in the adipose tissue can be observed (Victoria blue hematoxylin and eosin stain, original magnification ×40). These results are consistent with chronic panniculitis and intestinal wall fibrosis.

Laparoscopy revealed marked swelling of the rectum while the surrounding mesentery was sclerotic. Laparoscopic surgery was not feasible; therefore, the patient underwent laparotomy. The descending colon and surrounding mesentery were soft and showed no signs of inflammation. Partial rectal resection or Hartmann surgery was planned, but the risk of anastomotic leakage was high, and abdominal perineal resection was performed. Histopathological examination revealed narrowing of the venous lumen with marked thickening of the intima, even in the small veins and venules extending over the entire length of the intestinal tract, from the submucosa to the muscularis propria and submucosa. The arteries did not exhibit any intimal thickening (Figures 3A-3B), the vein wall did not show inflammatory cell infiltration, and there was no evidence of vasculitis. These results suggest that chronic elevation of intestinal venous pressure causes venous sclerosis and thrombosis (Figure 3C). Chronic lipofibrosis and fibrosis of the intestinal wall further triggered ischemic changes (Figure 3D). Based on these findings, ischemic colitis caused by venous congestion was diagnosed. The patient was discharged 18 days after surgery. No recurrence was noted at the one-year follow-up.

## Discussion

Ischemic colitis after colorectal surgery can be caused by venous congestion; in such cases, surgery may be required. As such, congestive ischemic colitis should be considered in cases where the mesenteric artery is preserved.

Ischemic colitis is caused by an inadequate supply of oxygenated blood to the intestinal tract owing to vascular damage. Most cases are transient and improve with conservative treatment involving resting the intestinal tract and administering antimicrobial agents. Nevertheless, ischemic colitis requires a prompt diagnosis and treatment to prevent necrosis [2,3].

Acute ischemic colitis is most commonly related to mesenteric artery embolism (50% of cases), mesenteric artery thrombosis (15%-25%), and non-obstructive mesenteric ischemia (20%-30%). Mesenteric venous thrombosis (3%-5%) rarely involves the colon [4,5], while chronic ischemic colitis is primarily related to atherosclerosis [2]. This condition may be caused by various factors, including circulatory failure, post-cardiovascular surgery, diabetes, dialysis, bacterial infection, adverse drug reactions, and trauma [6,7].

Postoperative complications of colorectal cancer surgery include suture failure, bowel obstruction, and surgical site infections [8]. Ischemic colitis may also occur after surgery for colorectal cancer. Postoperative colonic ischemia is primarily caused by inadequate arterial blood flow. Further, it has been reported that colonic ischemia occurs in 0.83% of patients with high ligation of the inferior mesenteric artery (IMA) for sigmoid colon and rectal cancer [9]. Adequate lymph node dissection for colorectal cancer patients has prognostic significance [10]. However, it is necessary to maintain arterial blood flow to prevent anastomotic leakage. Patients with low ligation (with preservation of the left colonic artery) experience fewer suture failures [1,10-12], safe and adequate lymph node dissection with no significant differences in the number of lymph nodes harvested [1,10-13], complications, 30-day mortality [1,11,14,15], and five-year survival rates [1,10-13]. Therefore, the mechanisms of ligation and preservation of the mesenteric arteries remain controversial [1].

Although ischemic colitis is generally caused by mesenteric venous thrombosis, resulting from blood flow stagnation, vascular injury, and hypercoagulability [4], it may also occur due to venous congestion after colorectal cancer surgery. In such cases, an imbalance between the arterial and venous blood flow in the remaining colon causes ischemic colitis after colorectal surgery. In addition, it is influenced by vascular factors such as diabetes mellitus, decreased intestinal peristalsis, and increased intestinal pressure [16]. In this case, the remaining intestinal tract was 12 cm from the peritoneal reflection, and the superior rectal artery (SRA) was preserved. It is technically difficult to cut off only the branches of the inferior mesenteric vein (IMV) and preserve the main trunk. The IMV was transected at the same level as the left colonic artery; however, ischemic colitis developed. The cause of the blood flow imbalance was unclear; however, an imbalance in the remaining intestinal tract occurred. Histopathological examination revealed ischemic colitis due to venous congestion. On the rectal side of the anastomosis, the arterial blood flow was maintained with IMA-SRA preservation, whereas the venous return was reduced with IMV ligation. This may have caused chronic venous congestion in the rectum, resulting in ischemic enteritis. Cases of diabetes mellitus-induced vascular damage have also been reported, and we believe that this caused the present case of congestive ischemic colitis.

Ischemic colitis due to venous congestion is usually managed with supportive care, such as bowel rest [2]; however, refractory cases require surgery [3]. It has further been reported that sufficient resection of the rectal side of the anastomosis is necessary in cases of preservation of the IMA to prevent ischemic colitis due to venous congestion after colorectal surgery. Resection should be performed in an area where the arterial and venous flows in the remaining colon are balanced [16].

## Conclusions

In conclusion, this case shows ischemic colitis caused by venous congestion may occur after colorectal surgery. In addition, there will be more cases of mesenteric artery preservation to prevent postoperative anastomotic leakage. Finally, attention should be paid to chronic ischemic colitis caused by venous congestion.
